# Theoretical and Experimental Analysis of Single-Arm Bimodal Plasmo-Photonic Refractive Index Sensors

**DOI:** 10.3390/s24123705

**Published:** 2024-06-07

**Authors:** Konstantinos Fotiadis, Evangelia Chatzianagnostou, Dimosthenis Spasopoulos, Stelios Simos, Dimitris V. Bellas, Omkar Bhalerao, Stephan Suckow, Max C. Lemme, Elefterios Lidorikis, Nikos Pleros

**Affiliations:** 1 Department of Informatics, Aristotle University of Thessaloniki, 54124 Thessaloniki, Greece; evachat@csd.auth.gr (E.C.); dspa@auth.gr (D.S.); simostyl@csd.auth.gr (S.S.); dbellas@uoi.gr (D.V.B.); 2 Center for Interdisciplinary Research and Innovation (CIRI-AUTH), Balkan Center, Buildings A & B, 10th km Thessaloniki-Thermi Rd, 57001 Thessaloniki, Greece; 3 Department of Materials Science and Engineering, University of Ioannina, 45110 Ioannina, Greece; elidorik@uoi.gr; 4 AMO GmbH, Advanced Microelectronic Center Aachen, 52074 Aachen, Germany; bhalerao@amo.de (O.B.); suckow@amo.de (S.S.); lemme@amo.de (M.C.L.); 5 Electronic Devices, RWTH Aachen University, 52074 Aachen, Germany

**Keywords:** refractive index sensor, sensitivity, co-integration, plasmonic, bimodal, SU-8 photonic waveguide, sensing area length

## Abstract

In this paper, we study both theoretically and experimentally the sensitivity of bimodal interferometric sensors where interference occurs between two plasmonic modes with different properties propagating in the same physical waveguide. In contrast to the well-known Mach–Zehnder interferometric (MZI) sensor, we show for the first time that the sensitivity of the bimodal sensor is independent of the sensing area length. This is validated by applying the theory to an integrated plasmo-photonic bimodal sensor that comprises an aluminum (Al) plasmonic stripe waveguide co-integrated between two accessible SU-8 photonic waveguides. A series of such bimodal sensors utilizing plasmonic stripes of different lengths were numerically simulated, demonstrating bulk refractive index (RI) sensitivities around 5700 nm/RIU for all sensor variants, confirming the theoretical results. The theoretical and numerical results were also validated experimentally through chip-level RI sensing experiments on three fabricated SU-8/Al bimodal sensors with plasmonic sensing lengths of 50, 75, and 100 μm. The obtained experimental RI sensitivities were found to be very close and equal to 4464, 4386, and 4362 nm/RIU, respectively, confirming that the sensing length has no effect on the bimodal sensor sensitivity. The above outcome alleviates the design and optical loss constraints, paving the way for more compact and powerful sensors that can achieve high sensitivity values at ultra-short sensing lengths.

## 1. Introduction

Nowadays, there is a great need for the development of novel refractive index (RI) sensors that can replace expensive and time-consuming laboratory techniques with fast and user-friendly portable devices. Among the technologies under consideration, integrated photonic sensors have attracted the interest of the scientific community [1,2,3,4], because they offer several advantages including (i) high sensitivity, (ii) real-time and label-free operation, and (iii) mass production at a low cost. Although photonic biosensors have already been commercialized, their sensitivity performance is inherently constrained by the partial exposure of the propagating mode to the sample under investigation, which necessitates the use of long sensor lengths in the range of several millimeters or even centimeters, severely impacting device compactness and the number of sensing elements that can be hosted on a single chip.

Plasmonic sensors have been proposed as an alternative sensing platform that can offer higher sensitivity values while retaining a short transducer length [5,6] as this takes advantage of the exposure of the entire plasmonic mode to the surrounding medium. On the other hand, the inevitable high propagation losses introduced by plasmonic structures can be partially counterbalanced by the selective co-integration of plasmonic segments in low-loss photonic integrated circuits, combining in this way the advantages of both technologies. So far, several works demonstrating plasmo-photonic sensors in either Mach–Zehnder interferometer (MZI) or ring configurations have been reported [7,8,9]. MZIs are more tolerant and flexible structures that can be easily engineered to achieve higher sensitivity values. However, the need for two spatially separated optical paths increases the required footprint and the calibration complexity, as both arms have to be configured for optimal interference conditions prior to the injection of the liquid analyte. In an attempt to miniaturize interferometric sensing devices, single-path interferometers that exploit modes with different properties propagating in the same physical channel, known as bi-modal interferometers, have been investigated [10,11,12,13,14,15]. Although these single-arm interferometric structures have been shown to provide moderate sensitivity values that can be valuable in certain applications, a compact and comprehensive theoretical model that can associate their sensitivity capabilities with the material and geometrical characteristics of the device is still missing, impeding in this way any sensor optimization concepts.

In this article, we extend our previous experimental work reported in [16] and present a holistic theoretical and experimental analysis of bimodal single-arm interferometric sensors, resulting in a closed mathematical expression for their sensitivity and its relation to sensor length and material platform. Based on this analysis, the sensitivity of a bimodal sensor turns out to be independent of the detection area length and depends only on the difference between the group indices of the two propagating modes. These findings are verified both numerically and experimentally using a plasmo-photonic bimodal sensor that employs an aluminum (Al) plasmonic stripe co-integrated on an SU-8 photonic platform. The length-independent sensing performance of the single-arm bimodal interferometer highlights an additional important advantage for this type of sensor, on top of their well-known single path and, as such, compact and higher-environmental-perturbation-tolerance properties: it relieves the need for sensitivity improvements because of the use of longer sensing lengths and higher sensor footprints. Instead, it can sustain higher sensitivities only by appropriately engineering the group indices of the two propagating modes while maintaining an ultra-short detection area. This can significantly contribute towards low optical losses by providing the flexibility of using shorter transducer lengths without sacrificing sensor sensitivity, which is especially critical when plasmonic sensing waveguides (WGs) are employed. 

## 2. Sensitivity Calculations

In principle, the bulk RI sensitivity (*S_b_*) of an optical sensor operating with spectral interrogation is defined by the amount of wavelength shift, $\Delta \lambda_{dip/peak}^{k}$, caused by the effective index change in the mode propagating along the sensing WG due to a change in the ambient RI. The sensitivity is expressed in units (nm/RIU). According to the analysis presented in [17], the wavelength shifts for an interferometric sensor, and consequently, sensitivity is described by the following compact equation:

(1) $$S_{b}=\Delta \lambda_{dip/peak}=\frac{L_{sen}\lambda_{dip/peak}}{\Delta (n_{g}L)}$$

where $L_{sen}$ represents the corresponding length of the sensing WG, $\lambda_{dip/peak}$ is the wavelength dip or peak, and Δ(ngL) for a conventional MZI is equal to

(2) $$\Delta (n_{g}L)=n_{g,ref}L_{ref}-n_{g,sen}L_{sen}$$

where $n_{g,ref}$ and $n_{g,sen}$ are the group indices of the modes propagating in the reference and the sensing arm, respectively, and $L_{ref}$ and $L_{sen}$ are the length of the reference and of the sensing arms, respectively. A bimodal configuration consists of one physical path and, for this reason, the length of the two optical paths is equal ($L_{ref}$ =$L_{sen}$).

So, Equation (2) is simplified to

(3) $$\Delta (n_{g}L)=L_{sen}(n_{g,ref}-n_{g,sen})$$

Replacing Equation (3) in (1), *S_b_* is calculated according to the following equation:

(4) $$S_{b}=\frac{\lambda_{dip/peak}}{n_{g,ref}-n_{g,sen}}$$

Equation (4) shows that the sensitivity of bimodal interferometric sensors is independent of the sensing area length. At first view, this result might seem counter-intuitive: by increasing the sensing length ($L_{sen}$) in an interferometric device, the accumulating phase shift of the mode propagating along the sensing WG increases, which increases the wavelength shift at the output and, thus, also the sensitivity. However, to explain the physical phenomenon behind this behavior, we should also consider that the amount of phase shift (in units of 2π) required for a unit wavelength shift, determined by the inverse of the FSR, also increases by the same proportion. Specifically, the equation that gives the FSR of the bimodal interferometer is

(5) $$\mathrm{FSR}=\frac{\lambda^{2}}{\Delta (n_{g}L)}=\frac{\lambda^{2}}{L_{sen}\Delta (n_{g})}$$

We observe that the FSR is inversely proportional to $L_{sen}$. Thus, the increase in the sensing length causes an FSR decrease, which reduces the sensitivity since a certain phase shift of the propagating mode now corresponds to a smaller wavelength shift (a full FSR corresponds to 2π phase shift). Consequently, sensitivity remains constant regardless of the sensing length. 

From another point of view, we can express the sensitivity as a function of FSR:

(6) $$S_{b}=\frac{\lambda }{\frac{\lambda^{2}}{L_{sen}\mathrm{FSR}}}=\frac{L_{sen}\mathrm{FSR}}{\lambda }$$

According to Equation (6), the sensitivity increases with increasing $L_{sen}$. However, according to Equation (5), the FSR decreases with increasing $L_{sen}$ which, in turn, decreases the sensitivity according to Equation (6). So, the sensing length has a contradictory effect on sensitivity, which finally remains constant irrespective of the sensing length. By changing the plasmonic stripe length, the surface RI sensitivity (*Ss*) will remain the same since *Ss* is calculated as follows:

(7) $$Ss=\frac{\delta n_{eff}}{\delta t}$$

where *n_eff_* represents the effective index of the top plasmonic mode waveguide and *t* is the thickness of the extra layer. *Ss* was calculated in our previous work and is presented in [16]. 

On the contrary, in a conventional MZI, the FSR is given by the following equation:

(8) $$\mathrm{FSR}=\frac{\lambda^{2}}{\Delta (n_{g}L)}=\frac{\lambda^{2}}{n_{g,ref}L_{ref}-n_{g,sen}L_{sen}}$$

And the sensitivity is given by

(9) $$S_{b}=\frac{L_{sen}\cdot \mathrm{FSR}}{\lambda }=\frac{L_{sen}\cdot \frac{\lambda^{2}}{n_{g,ref}\cdot L_{ref}-n_{g,sen}\cdot L_{sen}}}{\lambda }=>\;S_{b}=\frac{L_{sen}\cdot \lambda }{n_{g,ref}\cdot L_{ref}-n_{g,sen}\cdot L_{sen}}$$

So, in this case, the FSR depends not only on the length of the sensing arm but also on the length of the reference arm and their group index difference. Consequently, in the equation that gives the sensitivity, $L_{sen}$ is not eliminated and, thus, sensitivity increases with increasing $L_{sen}$.

## 3. Numerical Validation

**Figure 1:**
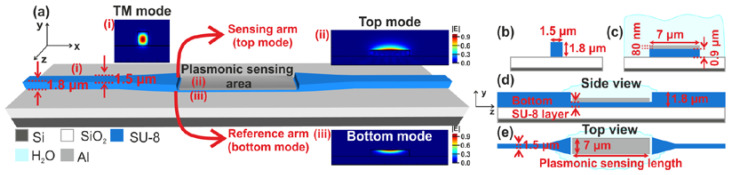
(**a**) Conceptual schematic of the bimodal refractive index sensor; and electric field distribution of the fundamental TM photonic mode (i), of the top mode (ii) and of the bottom mode (iii) (**b**) cross-section of the input SU-8 photonic WG with the fabricated dimensions; (**c**) cross-sectional dimensions of the aluminum plasmonic WG; (**d**,**e**) side view and top view of the presented device, respectively.

The integrated bimodal sensor under investigation consists of two photonic WGs based on the SU-8 material forming the input and output of the device, and an Al plasmonic stripe WG that is placed between them and on top of a suitably thinner SU-8 WG, called the bottom SU-8 layer. Figure 1a depicts the 3D conceptual schematic of the studied plasmo-photonic bimodal sensor, while Figure 1d,e show the side and top views of the structure, respectively. The plasmonic WG supports two different surface plasmon polariton (SPP) modes: (i) the top mode at the interface between the Al stripe and the surrounding medium which plays the role of the sensing branch, and (ii) the bottom mode at the interface between the Al stripe and the bottom SU-8 layer which plays the role of the reference branch. These modes are excited by a fundamental TM photonic mode launched into the input SU-8 WG and, after propagating along the metal surfaces, interfere at the output photonic part, realizing in this way the single-arm interferometer. 

A 1.8 μm × 1.5 μm (thickness × width) SU-8 (n_SU-8_ = 1.575 − 0.0001j) stripe WG core lying on top of a silicon oxide (SiO_2_) substrate was employed for the input and output photonic WGs. The WGs were not cladded and were exposed to the ambient. The cross-section of the photonic WG along with the supported TM mode profile are depicted in Figure 1b and Figure 1a(i), respectively. The plasmonic WG is formed by depositing an 80 nm × 7 μm aluminum (n_Al_ = 1.427 − 15.135j) [16] stripe on top of the bottom SU-8 layer with the same width. The cross-sectional dimensions of the Al-on-SU-8 WG along with the mode profiles of the top and bottom plasmonic modes are presented in Figure 1a(ii,iii),c. For the above dimensions and a bottom layer with 0.9 μm thickness, the effective and group indices obtained with 2D eigenvalue analysis are as follows: n_eff,top_ = 1.31 for the top and n_eff,bottom_ = 1.575 for the bottom mode, and n_group,top_ = 1.36 for the top and n_group,bottom_ = 1.62 for the bottom mode at λ = 1550 nm. The exact, optimum dimensions were defined based on a systematic design analysis targeting the (i) maximum RI sensitivity and (ii) maximum ER at the sensor output that allows lower detection limits. 

For the bimodal configuration, the Extinction Ratio (ER) is defined as

(10) $$\mathrm{ER}=10\log_{10}\left(\frac{T_{max}}{T_{min}}\right)$$

where *T_max_* and *T_min_* represent the maximum (peak) and minimum (dip) values of the power at the interferometer output, respectively. The holistic analysis of the ER calculation was presented in [16]. In order to achieve the maximum ER, the thickness of the bottom SU-8 layer was thoroughly investigated in combination with the appropriate plasmonic stripe length aiming at optimal interference conditions between the two plasmonic modes at the sensor output. The detailed design analysis is descripted in [16]. According to Equation (4), the theoretical sensitivity (*S_b_*) of the above bimodal sensor is 5961 nm/RIU at λ = 1550 nm.

**Figure 2:**
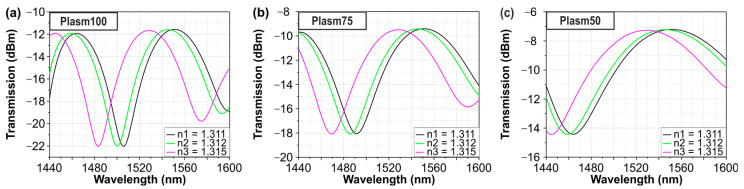
Simulated bimodal transfer function and sensitivity measurements for plasmonic length (**a**) 100 μm, (**b**) 75 μm and (**c**) 50 μm, respectively.

The numerical evaluation of the above bimodal sensor was conducted by means of circuit-level simulations using the INTERCONNECT photonic integrated circuit simulator of Ansys Lumerical. Three different structures with plasmonic lengths of 100, 75, and 50 μm were simulated in order to evaluate their sensitivity and how it is affected by the length of the plasmonic sensing area. Water cladding has been considered on top of the plasmonic stripe with an RI value obtained from the literature (n_water_ = 1.311 − 0.0001348j at 1550 nm [18]). The corresponding simulation curves are depicted in Figure 2a–c. As expected from the theory described above, by performing a linear least squares fit to the shift in the wavelength dip with the change in RI, the numerical sensitivity measurements revealed a bulk RI sensitivity of 5723 nm/RIU for all plasmonic lengths, validating that the sensitivity of the bimodal sensor is independent of the sensing length. 

## 4. Fabrication

**Figure 3:**
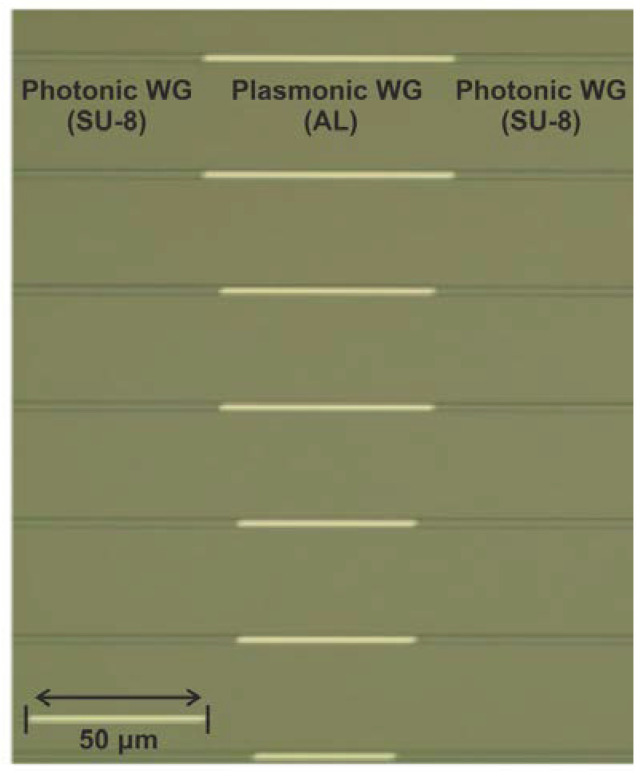
Top-view microscope image of fabricated plasmonic waveguides of different lengths.

The proposed sensor was fabricated on a 150 mm Si substrate containing a 3 μm thick thermally grown SiO_2_ layer. The first SU-8 layer was spin-coated on the entire wafer and a two-stage soft-bake process on hot-plates was followed. The SU-8 structures were exposed with optical i-line stepper lithography. After development, the wafer underwent a hard-bake process to cross-link the resist, resulting in the formation of the bottom SU-8 layer (referred to as the bottom SU-8 layer) with a measured thickness of 0.9 μm. The hard-bake process ensured the mechanical and chemical stability of the fabricated SU-8 WGs. After completing the fabrication of the bottom SU-8 layer, the Al layer was deposited using an electron beam evaporator, resulting in a plasmonic stripe WG. More specifically, a layer with 80 nm thickness was deposited onto the bottom SU-8 layer. Another i-line lithography process was used for the patterning of the metal layer. The process flow of the second (top) SU-8 layer was very similar to that described above. The height of the second SU-8 layer was equal to 0.9 μm. However, the deposition and exposure parameters were optimized considering the topography generated after the fabrication of the bottom SU-8 layer. Figure 3 depicts a microscope top-view image of the fabricated sensors showing varying Al lengths. To form the coupling edge required for butt-coupling, the wafer was diced down into 2 cm × 2 cm chips by gently sawing through the SU-8 WGs.

## 5. Experimental Results

**Figure 4:**
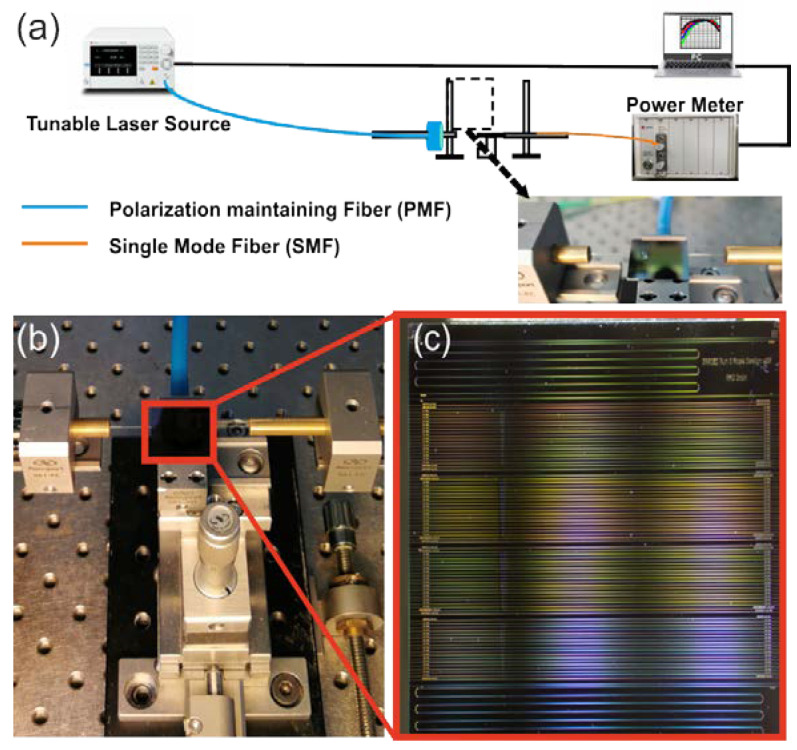
(**a**) Schematic of the experimental setup, (**b**) photo from the probe station used for optical characterization and refractive index experiments, (**c**) zoomed-in photo of the fabricated IC.

Figure 4a shows the experimental setup used to evaluate the sensors. The first step towards the optical characterization was measurements using a laser emitting at 640 nm and a visible camera to image the integrated plasmo-photonic sensors from above in order to more easily establish a clear optical link and, at the same time, to examine if the light propagates along the waveguides. The setup consists of the following components: a tunable laser source (TLS, Santec TSL 550, Higashisakura, Higashi-ku, Nagoya) that emits light from 1500 nm to 1630 nm with a resolution of 100 pm, a lensed polarization-maintaining fiber, integrated sensors with different plasmonic lengths, an optical power meter (MPM 211, Higashisakura, Higashi-ku, Nagoya), optomechanical components such as stages, piezoelectric controllers to accurately control the xyz stages in order to align the in/output fibers, and a fiber rotator to manipulate the inject polarization. Also, a vacuum chip holder, a vacuum pump, and a probe station were used in the experimental setup for stability, and finally a stereoscopic microscope was used during experiments. The light emitted by the TLS, through the lensed fiber at the input, was injected into the chip in TM polarity. The butt/edge coupling scheme was used for the optical in- and out-coupling. Figure 4b depicts a photo from the probe station used for optical characterization and RI sensing experiments, while Figure 4c presents a zoomed-in photo of the fabricated integrated plasmo-photonic sensors.

**Figure 5:**
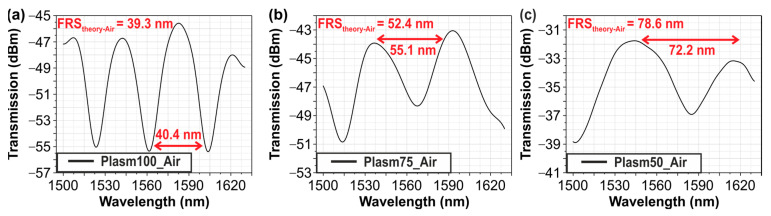
Experimental measurements versus wavelength for different plasmonic waveguide lengths in air condition, (**a**) 100 μm sensor, (**b**) 75 μm sensor and (**c**) 50 μm sensor.

The first characterization step to perform sensitivity measurements for the different plasmonic sensors was the optical characterization of the sensors in air. Figure 5a–c show the experimental spectra obtained for three different plasmonic lengths (i.e., 100, 75, and 50 μm). In each spectrum of the above plasmonic structures, a Fast Fourier Filter was applied, as mentioned also in [19], to better identify the interferometric response and track the performance of the structures. It is evident that the experimentally observed FSR values are in good agreement with the values expected from theory, confirming the bimodal operation. 

After completing the optical characterization of the sensors in air, the sensitivity experiments were conducted to evaluate the operation of the plasmo-photonic structures as an RI sensor. The sensitivity measurements were performed by spraying droplets of four different aqueous solutions over the plasmonic sensor region, as depicted in the inset in Figure 4. A commercial refractometer manufactured by Mettler Toledo 30 px company (Hamilton, Nea Zealand) was used to determine the RI of the samples, resulting in RI values between 1.3340 and 1.3573.

**Figure 6:**
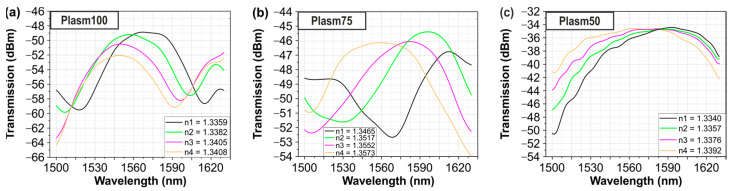
Experimental sensor spectral response when using different liquid samples with refractive index varying from 1.3340 to 1.3573 for different Al stripe length (**a**) 100 μm, (**b**) 75 μm and (**c**) 50 μm, respectively.

**Figure 7:**
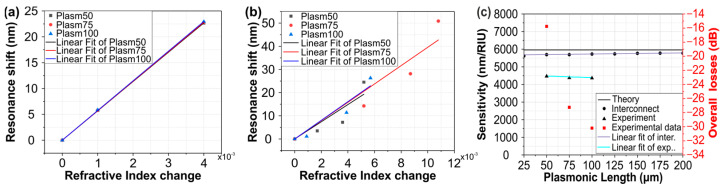
Resonance shift with respect to the refractive index change of the different solutions and least-squares linear fit for the different plasmonic waveguide lengths for (**a**) simulated data and (**b**) experimental measurements, (**c**) comparison in sensitivity values for different plasmonic length and extracting the data from mathematical equations, from interconnect and from experiment and the overall losses for different plasmonic stripe lengths.

Figure 6a–c present the smoothed measured sensor spectra when the sensor surface is covered by the liquid solutions, revealing a clear blue-shift in the resonance dip/peak with increasing RI values. Bulk sensitivity values were calculated for the resonance dip/peak by performing a linear least squares fit to the wavelength dip/peak with increasing RI values, as shown in Figure 7a,b for the experimental and simulated values, respectively. Using this method, we calculated the RI sensitivity for plasmonic lengths of 50, 75, and 100 μm and we obtained sensitivity values of 4464, 4386, and 4362 nm/RIU, respectively. The above experimental sensitivity values reveal strong agreement not only with the theory (5961 nm/RIU), but also with the corresponding value of about 5723 nm/RIU that was derived from the INTERCONNECT software (2022 R2.4). The left axis of Figure 7c depicts a comparison of the sensitivities between the experimental values, the values from INTERCONNECT, and the values expected from theory, while the right axis of the same figure presents the overall experimental losses of the sensor for different plasmonic WG lengths. Because the sensitivity in the theoretical analysis was calculated at λ = 1550 nm while, in INTERCONNECT, the dips/peaks were at slightly different wavelengths, there is a small difference in sensitivity values between the theoretical analysis and INTERCONNECT. Also, the deviation between the experimental sensitivity values and those from theory can be attributed to deviations in the geometrical and optical properties between the i‘deal’ simulated and the fabricated structures. The Linear Correlation coefficient for the simulated data (Figure 7a) is equal to 1 for all plasmonic stripe lengths and that for the experimental data (Figure 7b) are equal to 0.94, 0.95, and 0.85 for plasmonic stripe lengths equal to 100, 75, and 50 μm, respectively. According to Figure 7c, the sensitivity of the proposed sensor remains stable and does not depend on the detection area length, while the overall transmission losses reduce for shorter plasmonic WG lengths. A linear least squares fit was performed in the interconnect and experimental values of Figure 7c and the Linear Correlation coefficient was calculated and found to be equal to 0.94 and 0.85, respectively. Except for the sensitivity measurements, the figure of merit (FoM) of the proposed sensors was calculated. According to [20,21], the FoM is equal to

(11) $$\mathrm{FoM}=\frac{\mathrm{Sensitivity}}{\mathrm{FWHM}}$$

where FWHM is the full-width at half-maximum. Applying the above equation in the presented sensors, the FoM is equal to 175, 81, and 57 RIU^−1^ for 100, 75, and 50 μm plasmonic stripe lengths, respectively. For larger plasmonic stripe lengths, the FSR value is decreased, and the dip is narrower than in the case with smaller plasmonic stripe lengths and larger FSRs. So, the FWHM is smaller in sensors with longer plasmonic waveguides and, consequently, the FoM increases. 

Based on the above theoretical analysis, the experimental results presented in Figure 6, and the comparison in Figure 7c, the plasmonic length has no effect on the sensitivity of an interferometric sensor when based on a single-arm bimodal configuration. By excluding the plasmonic length as a parameter to increase the sensitivity, it is possible to increase the transmission with shorter stripe lengths, helping the signal-to-noise ratio and reducing system cost, leading to an improvement in the overall performance of the proposed sensor. Table 1 highlights the promise of the proposed bimodal plasmo-photonic sensor in offering high sensitivities within ultra-small sensing lengths in comparison to other, similar works.

## 6. Conclusions

We theoretically investigated the sensitivity of bimodal interferometric sensors, resulting in a mathematical equation for its calculation which shows that the sensing element length has no influence on their sensitivity. We validated the theory both numerically and experimentally by utilizing a plasmo-photonic interferometric bimodal sensor based on an Al-on-SU8 bimodal structure with lengths of 50, 75, and 100 μm. In all cases, the sensitivity remained almost constant, with the experimental (4464, 4386 and 4362 nm/RIU), numerical (5723 nm/RIU), and theoretical (5961 nm/RIU) values being in very good agreement. By proving that the sensitivity of bimodal interferometric sensors remains consistent across plasmonic sensors of different lengths, we open up new possibilities for ultra-compact and highly sensitive sensors, keeping the overall losses at a low level. 

## Figures and Tables

**Table 1:** A comparison table for the sensitivity value and the plasmonic stripe length between of some reported bimodal sensors.

Ref	Type	Sensitivity (nm/RIU)	Plasmonic Stripe Length (μm)
[8]	MZI	1930	70
[11]	MZI (bimodal)	2430	65
[15]	MZI (bimodal)	789	5000
[22]	SPR	683	500
**This work**	MZI (bimodal)	4362	50

## Data Availability

Please contact with the corresponding author (kfotiadi@csd.auth.gr) in order to found the reported results.
